# Updated data on the epidemiology of Inflammatory Bowel Disease (IBD) in Lebanon

**DOI:** 10.1371/journal.pone.0340892

**Published:** 2026-02-23

**Authors:** Antoine Abou Rached, Joyce Saniour, Antoine Geagea, Christian Salameh, Souheil Hallit, Charbel Yazbeck

**Affiliations:** 1 Faculty of Medicine, Lebanese University, Hadat, Lebanon; 2 School of Medicine and Medical Sciences, Holy Spirit University of Kaslik, Jounieh, Lebanon; 3 Applied Science Research Center, Applied Science Private University, Amman, Jordan; 4 Department of Gastroenterology, Notre-Dame des Secours Hospital, Byblos, Lebanon; Independent Medical Researcher and Writer, UNITED KINGDOM OF GREAT BRITAIN AND NORTHERN IRELAND

## Abstract

**Objective:**

The objective of the study was to review available data on IBD in Lebanon based on histopathological reports and endoscopic data to identify the number of newly diagnosed cases per year, age, gender, and disease phenotype, and subsequently update the incidence and prevalence of the disease.

**Methods:**

A review of all histopathological reports of newly diagnosed IBD patients over a 20-year period was conducted, along with a review of available clinical and endoscopic information at the National Institute of Pathology. The clinical data analysed included the type of IBD, age, gender, number of cases diagnosed per year, location of each IBD type and disease progression over time. The Montreal classification for age and phenotype was applied to both diseases.

**Results:**

Over a 20-year period (from January 2000 to December 2020), 2,869 IBD patients (1,365 with Crohn’s disease (CD) and 1,504 with ulcerative colitis (UC) were included. Our sample represents 20% of the Lebanese population with IBD. No cases of unclassified IBD were identified since all patients were followed up throughout the years. The male-to-female ratio was 1.15 for UC patients and 1.3 for CD patients. The average age was 41 years for UC and 36.4 years for CD. Ileal involvement and left-sided colonic involvement were the most frequently noted in CD and UC, respectively. A progressive increase in diagnosed cases of CD and UC was observed over this 20-year period. The annual incidence rose from 4.9 to 11 per 100,000, with a cumulative incidence of 6.53 per 100,000 and a prevalence of 130.56 per 100,000. The highest incidence occurred between ages 21–30. Incidence trends were independently predicted by age group and calendar period, with markedly higher rates among individuals aged 20–39 and 40–59 years with a significant increase in the 2015–2020 period, whereas sex and disease type showed no significant associations.

**Conclusion:**

Epidemiological data demonstrated a progressive increase in the incidence of IBD over this 20-year period, with a slight male predominance. Ileal involvement is predominant in CD, whereas involvement extending beyond the splenic flexure is present in more than half of UC cases.

## Introduction

Inflammatory bowel disease (IBD), a chronic and relapsing group of conditions affecting the gastrointestinal tract, poses a significant challenge for healthcare professionals worldwide due to its rising prevalence, clinical heterogeneity, and substantial impact on patients’ quality of life [1,2]. Characterized by chronic inflammation of the digestive tract, primarily in the forms of Crohn’s disease (CD) and ulcerative colitis (UC), IBD manifests through symptoms such as abdominal pain, diarrhea, rectal bleeding, and weight loss, with episodes varying in severity and duration [3,4]. The pathogenesis is multifactorial and encompasses genetic susceptibility, mucosal barrier dysfunction, dysregulated immune system, and environmental influences (such as diet, smoking and microbiome alterations) [5].

Globally, the burden of IBD has increased substantially over recent decades [2]. Systematic reviews demonstrated that more than 4.9 million individuals worldwide were living with IBD, in 2019 [5,6]. Those trends are increasing. Projections suggest that by 2030, more than 7 million people in Europe and the United States will be affected by IBD [7]. This rising burden imposes direct medical costs (hospitalisations, surgery, long-term medication) and indirect costs (loss of productivity, absenteeism), creating a considerable economic and public health challenge [5,7].

IBD prevalence, clinical presentation, course and treatment vary with sex, age and disease location [7–11]. CD is slightly more common in women, especially in Western countries [8,9]. For UC, the incidence is generally similar between sexes before age 45; however, after 45, males demonstrate a higher risk [10]. The CDC reported higher percentages in adults aged 45–64 (1.5%) and ≥65 (1.7%) compared to younger age groups [11]. Despite increasing prevalence in some countries, others have different trends. Inflammatory bowel diseases (IBD) were first described in Northern Europe and the United States. Although initially confined to these regions, IBD has now emerged globally, with incidence and prevalence varying significantly. In Europe, UC incidence ranges from 0.9 to 24.0 per 100,000 people, while CD incidence varies from 0.0 to 11.5 per 100,000 [12,13]. The prevalence of UC and CD in Europe spans 2.4 to 294 and 1.5 to 213 cases per 100,000, respectively [14–16]. North America exhibits similarly high rates, with CD and UC incidence ranging from 0 to 20.2 and 0 to 19.2 per 100,000 people, respectively [17]. Prevalence rates reach up to 318.5 per 100,000 for CD and 248.6 per 100,000 for UC [18,19]. Outside of Europe and North America, traditionally low-incidence regions such as Asia and South America have witnessed a rapid rise in IBD cases, likely driven by urbanization and lifestyle changes [20]. Recent meta-analyses report that the incidence of UC is highest in Europe (24.3 per 100,000) and North America (19.2 per 100,000). In contrast, the incidence of CD is highest in Asia (20.2 per 100,000), followed by Europe (12 per 100,000) [21]. In developing nations, IBD incidence shows that IBD remains sporadic. However, newly industrialized regions demonstrate substantial increases in incidence, signifying a growing socioeconomic impact [22,23].

Hence, the epidemiology of IBD can be categorized into four stages: emergence, incidence acceleration, compounding prevalence, and prevalence equilibrium [21]. Developing nations remain in the emergence stage, characterized by sporadic cases [24,25]. The chronic and progressive nature of IBD, coupled with the high costs of treatment, underscores the growing burden posed by its rising incidence.

In Arab nations, including Lebanon, IBD data remains sparse. However, recent studies indicate a sharp rise in incidence, paralleling global patterns [24,26,27]. In Lebanon, the preliminary insights provided useful early insights about prevalence trends [24], but those estimates were derived from a relatively small, university affiliated, clinical registry records of mainly insured people over a limited timeframe from 2000–2004 and relied heavily on administrative and clinical records. These methodological constraints limit generalizability and the ability to assess long-term incidence and prevalence trends.

Our study addresses the gaps in the literature by using pathology-confirmed IBD diagnoses, larger scale across a 20-year interval. This study aimed to update the epidemiological and clinical characteristics of CD and UC in Lebanon, focusing on sex ratio, age at diagnosis, and disease location. It sought to calculate cumulative incidence and prevalence, highlighting changes in epidemiology and clinical features over a 20-year period. This approach increases case ascertainment validity and paves the way for temporal and subgroup analyses essential for public health planning including resource allocation, earlier diagnosis, and national surveillance to potentially reduce the clinical and economic burden of IBD in Lebanon.

## Methods

### Ethics approval and consent to participate

The study protocol was approved by the ethics committee of the Notre Dame des Secours University Hospital. All methods were carried out in accordance with relevant guidelines and regulations.

### Study design and data source

This multicentred, retrospective chart review study analysed data from the Lebanese National Pathology Institute between July 4 and August 10, 2025, representing 20% of all Lebanese pathology specimens from January 2000 to December 2020 [27].

### Population and case identification

Patients included in this study had a confirmed IBD diagnosis based on clinical and pathological findings. All Lebanese patients of any age with confirmed newly IBD diagnosis based on clinical and pathological findings were included. Non-Lebanese patients were excluded and patients known to have IBD before starting of the study. All Lebanese patients of any age with confirmed newly IBD diagnosis based on clinical and pathological findings were included. Non-Lebanese patients were excluded and patients known to have IBD before starting of the study.

### Data collection and analysis

Data collected included: year of diagnosis, age, gender, and UC/CD phenotype. The Montreal classification [27] was applied to categorize age at diagnosis and disease phenotype.

### Statistical analysis

The diagnosis of IBD was based on histopathology in conjunction with available endoscopic/clinical information at the National Pathology Institute. Histological features supporting IBD included chronic active colitis/ileitis with compatible patterns such as crypt architectural distortion, basal plasmacytosis, chronic inflammatory infiltrates, cryptitis/crypt abscesses, and/or ulceration; features supporting Crohn’s disease included focal/discontinuous (patchy) inflammation, transmural lymphoid aggregates when assessable, and/or non-caseating granulomas when present. Cases were classified as ulcerative colitis (UC) or Crohn’s disease (CD) based on the integrated pathology report and the clinical/endoscopic data available; no indeterminate/unclassified IBD remained after follow-up review in the institute records.

The main study variables were: year of diagnosis, age at diagnosis, sex, IBD type (UC/CD), and phenotype measures according to the Montreal classification. Montreal classification was applied as follows:

- Age at diagnosis divided into categories of 10 years.
- CD location: L1 (ileal), L2 (colonic), L3 (ileocolonic), L4 (isolated upper gastrointestinal involvement) when documented.
- UC extent (when available from endoscopy/pathology summaries): E1 (proctitis), E2 (left-sided colitis), E3 (extensive colitis) (presented in figures/tables as in the manuscript).

Categorical variables were summarised as numbers and percentages; continuous variables were summarised as mean (SD) or median (IQR) depending on distribution. Normality of continuous variables was assessed using graphical methods (histograms and Q–Q plots) and the Shapiro–Wilk test. Between-group comparisons used Chi-square or Fisher’s exact tests for categorical variables. For continuous variables, Student’s t-test was used for approximately normally distributed variables; otherwise, Mann–Whitney U test was used. All tests were two-sided, with α = 0.05; 95% confidence intervals (CIs) were computed for key estimates.

Incidence and prevalence calculations: annual incident cases were defined as the number of newly diagnosed pathology-confirmed IBD cases per calendar year. Cumulative incidence over the study interval was calculated as the total number of newly diagnosed cases during 2000–2020 divided by the corresponding population denominator and expressed per 100,000. Prevalence was reported as a period prevalence over 2000–2020 (total pathology-confirmed IBD cases identified during the interval divided by the population denominator, per 100,000), because consistent annual denominators and the requirements for estimating point prevalence were not available for all years in this dataset.

Data entry and analysis were conducted using R software, version 4.5.1. Categorical variables were analysed using the Chi-square test or Fisher’s exact test. Results were presented as numbers, percentages, and means. For all comparisons, 95% confidence intervals (CIs) were computed for means and proportions. Comparison between sex, age categories and groups of different location of each disease was performed. The disease location is categorized into four types: L1 (ileal), L2 (colonic), L3 (ileocolonic), and L4 (isolated upper gastrointestinal involvement).

To ensure reproducibility, all R packages used in data management, modelling, and visualization are cited according to the recommended references. Graphical outputs were generated using *ggplot2 [*28*]*. Data were handled using functions from *dplyr [*29*]* and *readr [*30*]*. Poisson and quasi-Poisson regression models were fitted using the stats package in base R [31]. For the APC analysis, robust standard errors were obtained using the *sandwich package [*32*].*

To evaluate temporal trends in incidence, we fitted a Poisson regression model with year entered as a continuous predictor. The Annual Percentage Change (APC) was computed using the standard formula:

$\text{APC }={(e}^{\beta_{\text{1}}}-\text{1}\times \text{1}\text{0}\text{0}$,

where $\beta_{\text{1}}\;$is the regression coefficient for year. Because the model exhibited overdispersion (Pearson χ²/df = 13.37), a quasi-Poisson model with robust standard errors was applied. APC estimates are reported with 95% confidence intervals.

Model adequacy was assessed by examining the residual deviance, residual degrees of freedom, and the dispersion parameter (ϕ) to evaluate over dispersion and overall goodness of fit. Residual independence was further assessed by inspecting the autocorrelation function (ACF) of Pearson residuals, which did not reveal meaningful serial autocorrelation.

To examine potential effect modification, interaction terms (sex x age group and sex x period) were tested by extending the Poisson regression models. Neither interaction term was statistically significant, and inclusion of these terms did not improve model fit; therefore, only main effects were retained in the final model. No offset term was included because models were applied to counts of pathology-confirmed cases rather than population-based incidence rates, and stratified population denominators (by age, sex and period) were not available to define a reliable offset.

## Results

### General characteristics of the population

Missing data for age were as follows: 1 out of 1,466 for ulcerative colitis (UC) and 139 out of 1,365 cases for Crohn’s disease. Regarding sex, there were no missing data. For disease location, missing data were observed in 3 out of 1,365 in CD and 4 out of 1,466 in UC.

Between January 2000 and December 2020, a total of 2,831 patients were diagnosed with IBD, including 1,466 cases of ulcerative colitis (UC; 51.8%) and 1,365 cases of Crohn’s disease (CD; 48.2%). Among patients with CD, 771 (56.5% [95% CO 53.81%−59.13%]) were male and 594 (43.5%) were female, yielding a sex ratio of 1.30 in favour of males. The mean age at diagnosis for CD was 36.35 years (95% CI: 35.49–37.21; SD 15.30, min = 2, max = 85). For UC, 785 patients (53.5% [95% CI 50.95%−56.12%]) were male and 681 (46.5%) females, corresponding to a sex ratio of 1.15 in favour of males. The mean age at diagnosis for UC was 35.74 years (95% CI: 34.61–36.87; SD: 22.02, min = 0, max = 93).

There was no significant difference between UC and CD in terms of gender distribution, X^2^(1) = 2.46, p = 0.121, nor in mean age at diagnosis, t(2689)= 0.84, p = 0.398.

**Fig 1:**
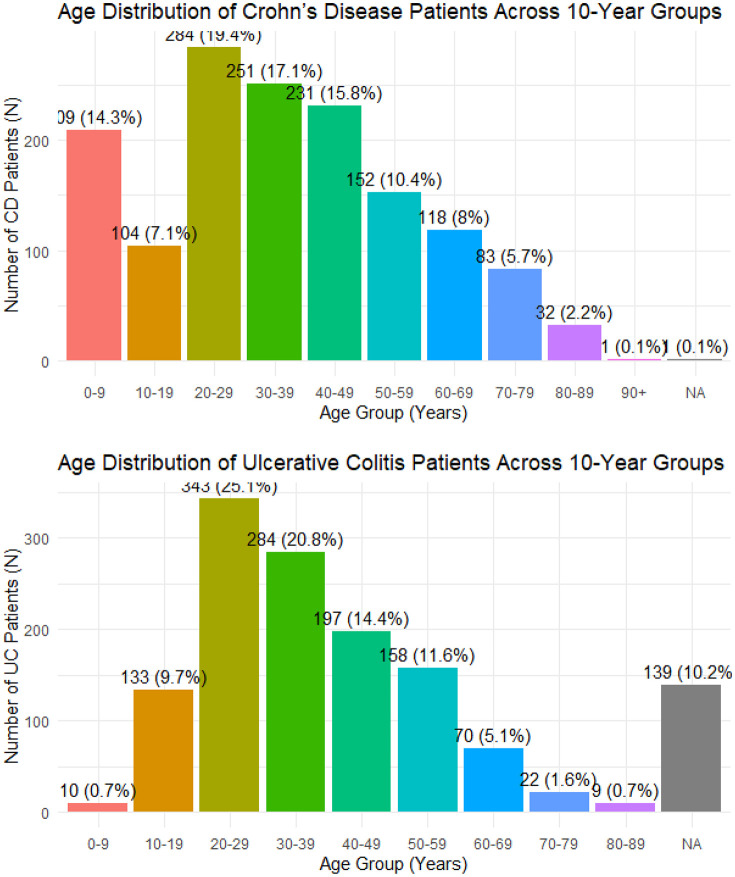
Age distribution of patients with Crohn’s disease and ulcerative colitis across 10-year age groups. a. Age distribution across 10-year age groups for patients with Crohn’s disease (CD). b. Age distribution across 10-year age groups for patients with ulcerative colitis.

**Fig 2:**
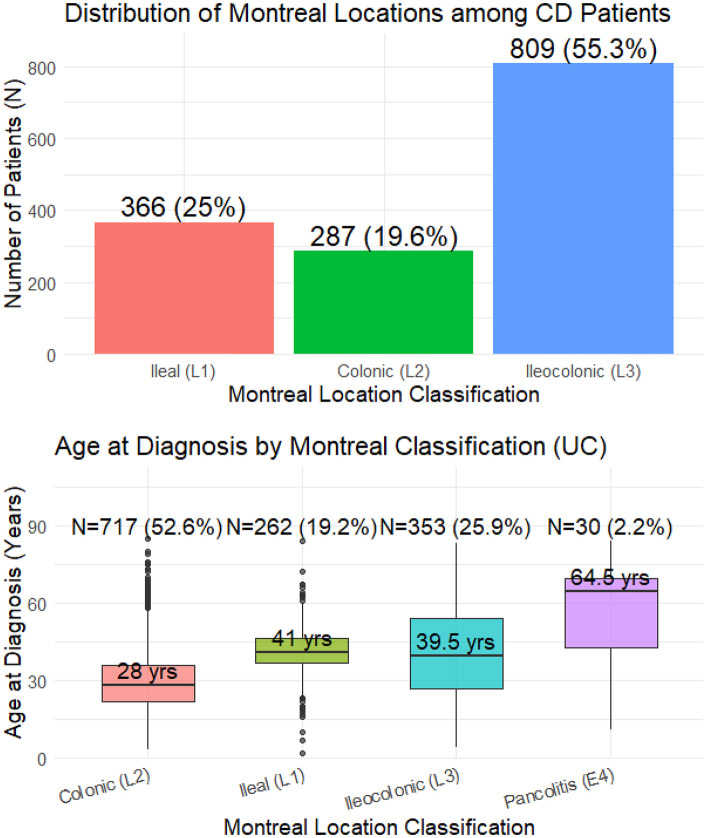
Age distribution by Montreal classification for Crohn’s disease and ulcerative colitis. a. Age distribution by Montreal classification in Crohn’s disease (CD). b. Age distribution by Montreal classification in ulcerative colitis (UC).

The age distribution of patients with UC and CD per 10-year age groups is represented in Fig 1a and 1b and according to Montreal classification in Fig 2a and 2b. For Crohn’s disease, the age distribution shows that most diagnoses occur between 20–39 years, peaking in the 20–29 years age group (n = 284). Median age at diagnosis is relatively consistent across Montreal locations (37 years for colonic (L2), 35 years for ileal (L1) and 34 years for ileocolonic (L3)), indicating that CD tends to affect young adults regardless of disease location.

For UC, the diagnosis is also most common in young adulthood, particularly in the 20–39 years groups (n = 627 combined). Median ages differ substantially across Montreal classification categoriess (28 years for ileal (L1), 39 years for ileocolonic (L3), 41 years for colonic (L2) and a much higher median age 64.5 years for pancolitis (E4)), suggesting that extensive UC pancolitis) is more frequent in older ages.

**Fig 3:**
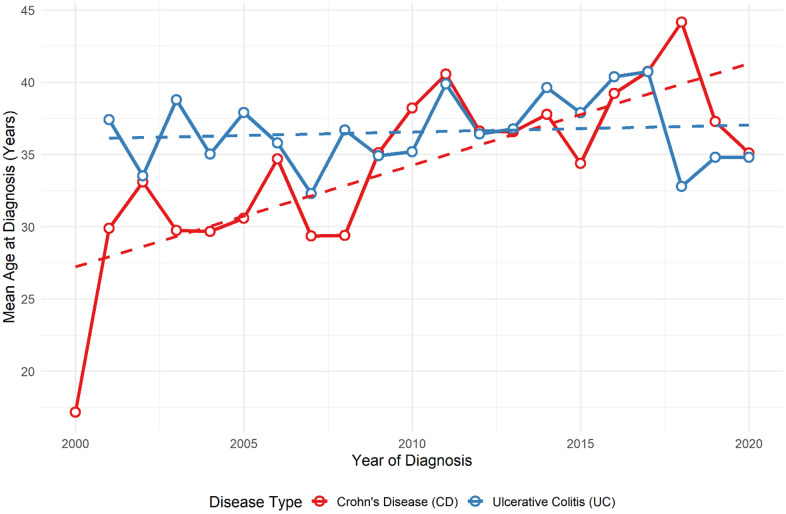
Evolution of mean ages of patients with CD and UC during the 20 year study-period. Solid lines represent the annual mean age for each disease group (red for CD and blue for UC). Dotted lines represent the fitted linear trend lines for each disease type, illustrating changes over time.

Fig 3 illustrates the change in mean age at diagnosis for CD and UC between 2000 and 2020. For CD (red line), the mean age shows a gradual increase from 25 years in 2000 to nearly 38 years in 2020. In contrast, the mean age for UC (blue line) remains relatively stable around 35–38 years throughout the period. The trend lines confirm an upward slope for CD and a flat slope for UC, indicating that diagnosis age has shifted upward for CD but not UC.

### Location distribution per age group and gender of patients with UC and CD

**Fig 4:**
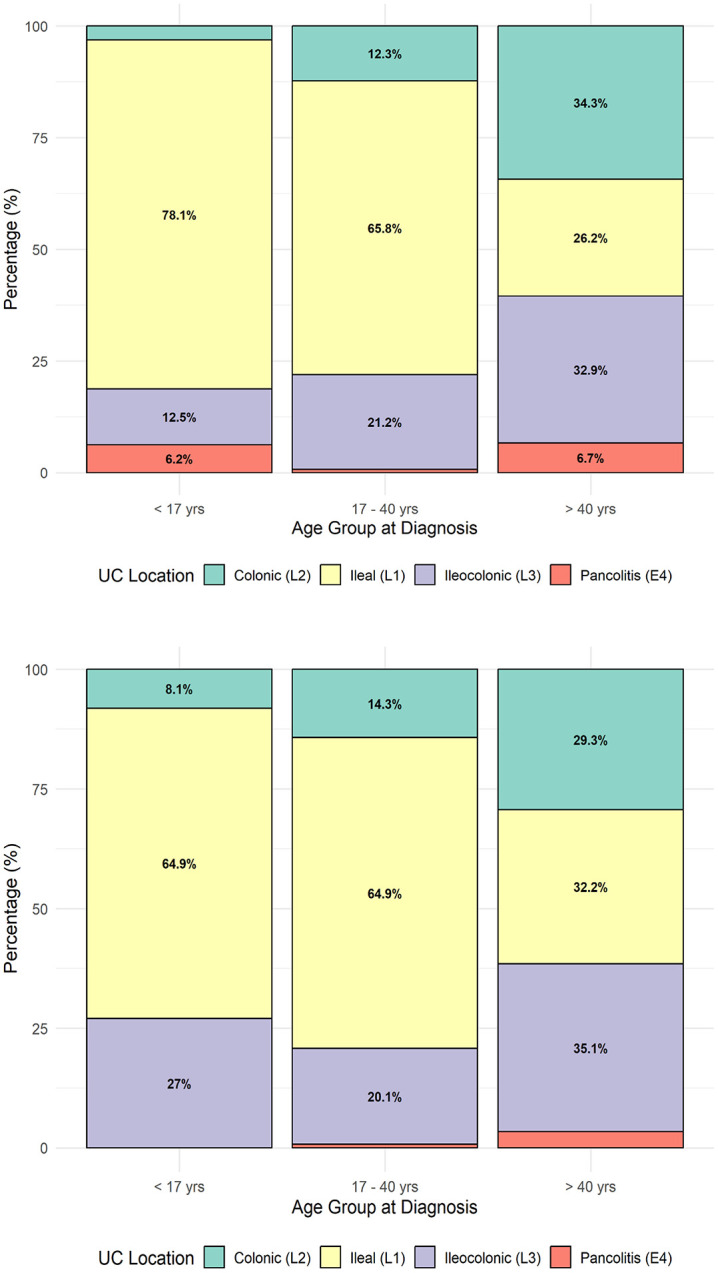
Location distribution across age groups for ulcerative colitis by sex. a. Location distribution across age groups in males with UC. b. Location distribution across age groups in females with UC.

For UC, the distribution shifts considerably with age. In both males and females, ileal involvement dominates in patients < 17 years, accounting for around 65–78% of cases, but declines steadily in older groups. With advancing age, colonic and pancolitis forms become more prevalent, reaching about 26–35% for distal and 6–7% for pancolitis in males over 40 years, and 29–35% and around 6% in females. This pattern suggests that UC tends to present as more extensive disease with increasing age at diagnosis, particularly among males (Figs 4a and 4b).

**Fig 5:**
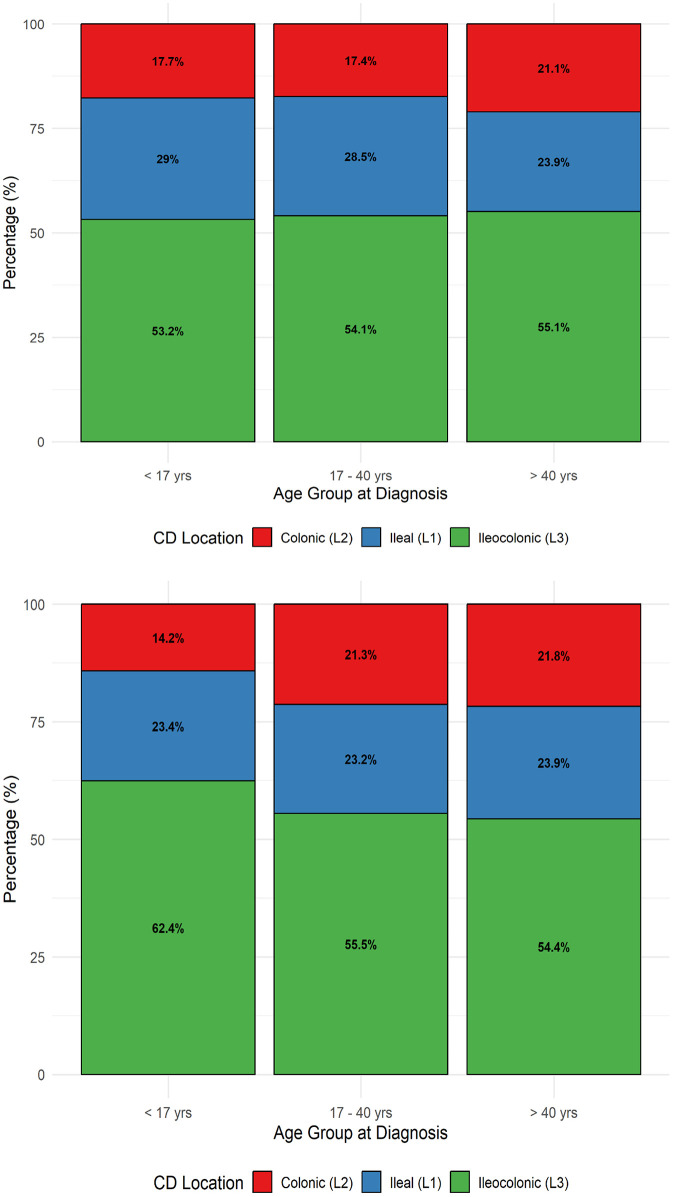
Location distribution across age groups for Crohn’s disease by sex. a. Location distribution across age groups in males with CD. b. Location distribution across age groups in females with CD.

For CD, the ileocolonic location predominates in both sexes and across all age groups, affecting about 53–55% of males and 54–62% of females. The ileal form represents roughly 24–29% of cases, while colonic disease is less frequent (about 17–21%). These proportions remain fairly stable across age groups, indicating that disease extent at diagnosis does not vary substantially with age or sex in CD, though colonic involvement appears slightly higher in older males and females (Figs 5a and 5b).

### Incidence of IBD in Lebanon

**Fig 6:**
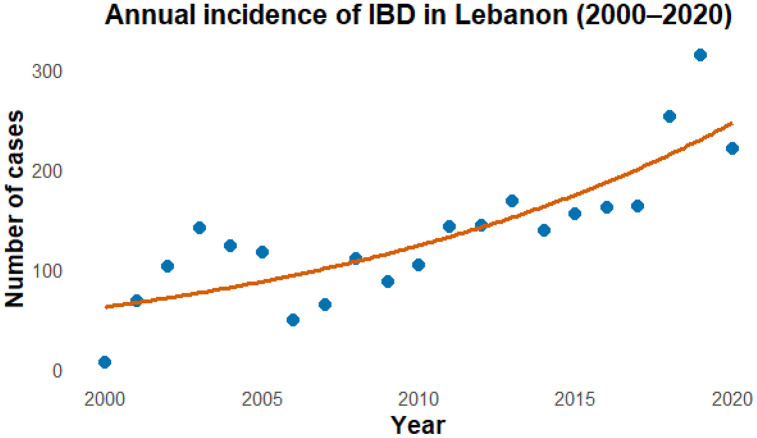
Annual incidence of IBD in Lebanon from 2000 to 2020. Blue points represent the observed annual number of new IBD cases, and the orange line represents the fitted Poisson regression model using u = year as a continuous predictor.

Fig 6 illustrates the annual incidence of IBD in Lebanon from 2000 to 2025, based on observed data and a fitted Poisson regression trend. The results show a marked upward trajectory in new IBD cases over time, with incidence rising from fewer than 50 cases per year in the early 2000s to over 300 cases by 2019, followed by a slight decline afterwards. The fitted Poisson trend line (gray) confirms a steady exponential increase, indicating a sustained rise in IBD incidence across the two decades. This pattern reflects the growing burden of IBD in Lebanon.

### Cumulative incidence and prevalence

Across the 20-year study period (2000–2020), the cumulative incidence of IBD was 6.53 per 100,000 population. The annual incidence increased from 4.9 per 100,000 in 2000–11 per 100,000 by 2020. The overall prevalence of IBD in Lebanon was estimated at 130.56 per 100,000 population. This estimate corresponds to a 20-year period prevalence, defined as the total number of pathology-confirmed IBD cases identified between January 2000 and December 2020, as annual population denominators were not consistently available to compute point prevalence. Prevalence trends mirrored the incidence pattern, showing a progressive increase throughout the study period, consistent with the growing number of diagnosed cases and the chronic nature of IBD.

### Annual Percentage Change (APC)

**Fig 7:**
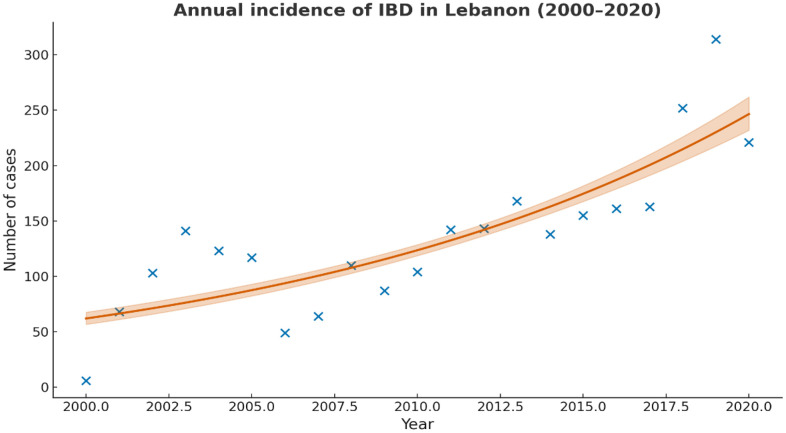
Annual incidence of inflammatory bowel disease (IBD) in Lebanon from 2000 to 2020. The observed yearly counts are shown as points. The solid curve represents the fitted quasi-Poisson regression model using year as a continuous predictor, and the shaded area indicates the corresponding 95% confidence interval. Based on this model, the estimated Annual Percentage Change (APC) in IBD incidence was 7.1% per year (95% CI: 4.7-9.7).

The incidence of IBD increased by 7.1% per year (95% CI: 4.7–9.7, p < 0.001) over the study period (Fig 7). Inspection of Pearson residual autocorrelation showed no meaningful serial autocorrelation, supporting the adequacy of the quasi-Poisson model. Goodness-of-fit diagnostics indicated a dispersion parameter of ϕ = 13.37, with a residual deviance of 275.70 on 19 degrees of freedom, consistent with the present of over dispersion handled by the quasi-Poisson specification.

### Relative measures (IRRs)

**Fig 8:**
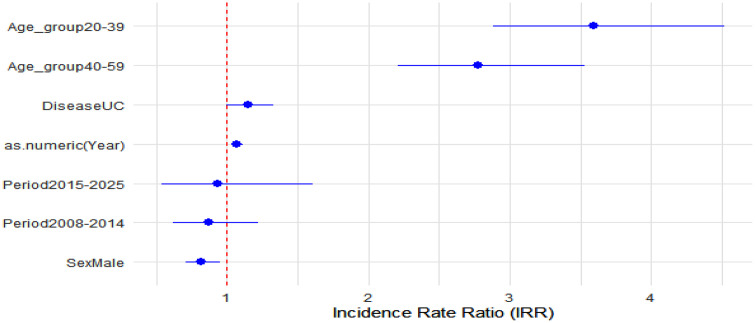
Forest plot of incidence rate ratios (IRRs) with 95% confidence intervals for sex, age groups, disease type, and calendar periods, Lebanon 2000-2025. The vertical dashed line incidicates the null value (IIR = 1).

We extended the Poisson regression to include sex, age groups, disease type, and calendar periods as predictors. We report incidence rate ratios (IRRs) with 95% CIs to quantify relative differences between groups. Key findings include: higher incidence in individuals aged 20–39 (IRR = 4.40, 95% CI:3.17–6.10) and 40–59 (IRR = 2.80, 95% CI:2.04–3.84) compared to <20; a significant increase in 2015–2025 compared to 2000–2007 (IRR = 2.30, 95% CI: 1.68–3.16) (Table 1 and Fig 8).

**Table 1:** Incidence Rate Ratios (IRRs) for IBD incidence, Lebanon 2000-2025.

Predictor	IRR	95% CI lower	95% CI upper	*p*-value
Sex: Male	1.22	0.95	1.56	0.113
Age group: 20–39	4.40	3.17	6.10	<0.001
Age group: 40–59	2.80	2.04	3.84	<0.001
Age group: 60+	1.27	0.91	1.78	0.166
Disease: UC	0.96	0.75	1.23	0.761
Period: 2008–2014	1.51	1.10	2.08	0.011
Period: 2015–2025	2.30	1.68	3.16	<0.001
Year (continuous)	1.09	1.06	1.11	<0.001

Neither sex x age group nor sex x period interaction terms were statistically significant, and thus were not retained in the final model.

## Discussion

Over the 20-year study period (2000–2020), we identified a total of 2,869 IBD patients in Lebanon (1,365 with Crohn’s disease and 1,504 with ulcerative colitis), representing approximately 20% of the nation’s IBD population. A clear upward trend in IBD burden was observed: the annual incidence of IBD more than doubled from 4.9 per 100,000 at the beginning of the study to about 11 per 100,000 by 2020. This corresponded to a cumulative incidence of 6.53 per 100,000 over two decades, alongside an overall prevalence of roughly 130.56 per 100,000. The highest incidence was noted in young adults (ages 21–30 years). The cohort’s demographic profile showed a slight male predominance in both UC (male-to-female ratio ~1.15) and CD (~1.3). The mean age at diagnosis was 41 years for UC and 36 years for CD. In terms of disease phenotype, CD cases were predominantly ileal, whereas UC cases most often presented with extensive colitis (disease extending beyond the splenic flexure). Collectively, these findings indicate a progressively rising incidence of IBD in Lebanon accompanied by notable patterns in age of onset, sex distribution, and disease extent.

### Age at diagnosis

In Lebanon, the mean age at diagnosis for UC and CD was 41.05 and 36.35 years, respectively, with a decrease in the mean age at diagnosis over 20 years. In comparison, Northern Europe, North America, and Oceania report an age of IBD onset of 31–34 years, while in Asia, CD and UC median ages are 34 and 42 years, respectively [20,21,33,34]. It is well known that the diagnosis of IBD can occur at any age [12,13,35–38]. A peak in the incidence of CD occurred between the ages of 20 and 30, while in UC, the peak occurred between 30 and 40 years of age. Additionally, a second, smaller peak in UC incidence was reported in individuals in their 60s and 70s [38–40].

### Sex distribution

In our Lebanese cohort, a male predominance was observed in both UC and CD, with a more pronounced ratio in CD (1.3) compared to UC (1.15). In contrast, Europe, North America, and Oceania demonstrated no gender difference in UC incidence. Some Western studies report a female predominance in CD, while others find no sex difference [35,41–44]. Conversely, in Asia, men are at a higher risk of both CD and UC, with a relative risk of 1.15:1 and 2.4:1, respectively, as seen in China, with similar findings in Korea [44]. A meta-analysis in Asia also highlights a higher CD and UC risk in males aged 10–50 years [45]. Previous studies in Saudi Arabia [46], Kuwait [47] and Lebanon also report a slight male predominance in UC.

### Disease phenotype

In Lebanon, extensive colitis is the most common form across age groups in UC patients. In UC, the disease extent at the time of diagnosis is roughly similar across different populations, with some variations in the rates of proctitis. Approximately one-third of patients will present with disease limited to the rectum (proctitis), one-third with involvement limited to the portion of the colorectum distal to the splenic flexure (left-sided colitis), and one-third with involvement proximal to the splenic flexure (extensive colitis) [12,35,38,48].

In line with European and North American data, Lebanese patients with CD predominantly exhibit ileal involvement, with a more balanced distribution across the three phenotypes (L1, L2, L3) in patients older than 40 years. In Western studies, 27–42% of patients with CD diagnosis presented with ileal location, 28–35% with colonic location, and 23–33% with ileocolonic location [44,49,50], while 1–6% had isolated upper gastrointestinal disease. Notably, New Zealand reports a higher prevalence of upper gastrointestinal involvement at 22%, while in Asia, 50–61% of CD patients predominantly show ileocolonic involvement [20,51].

Western data indicate that 23–34% of UC patients present with proctitis and 34–51% with left-sided colitis [35,49,51,52], with similar findings in Asia. Around 30–38% of Western UC patients show extensive colitis at diagnosis compared to 20–31% in East Asia.

### Incidence and prevalence

During the 20-year period, we noted a steady increase in the incidence of UC and CD, confirming that Lebanon is in the phase of acceleration in IBD incidence. The rising incidence and prevalence of IBD in Lebanon mirror patterns observed in other newly industrialized regions such as East Asia and South America, where urbanization and lifestyle changes have contributed to a significant increase in IBD cases [53]. In Asia, the prevalence of IBD is still low when compared to prevalence in the West [54,55]. For instance, in Europe, the highest prevalence of UC and CD were 505 and 322 per 100,000, respectively, while in North America, they were 249 and 319 per 100,000 people [55]. In contrast, the prevalence rates in Asia are significantly lower, with the highest reported rates being 168 per 100,000 for UC and 68 per 100,000 for CD [55]. However, the IBD population in Asian countries is expanding rapidly with the rising incidence [33,34,56].

Overall, 0.3% of the European population are estimated to have been diagnosed with IBD, corresponding to a total of 2.5–3 million IBD patients [57].

In a recent systematic review, concerning the epidemiology of IBD in Arab countries, the authors found that the incidence of IBD is increasing exponentially in most of the Arab countries, with an estimated incidence of UC and CD approximating 2.33 and 1.46 per 100,000 persons per year, respectively.

Unlike Western countries with well-established healthcare resources for CD management, Lebanon’s healthcare system faces significant challenges in meeting the needs of a growing IBD population, highlighting the necessity for public health interventions and resource allocation.

### Limitations and strengths

This study provides valuable epidemiological insights into IBD in Lebanon, but several limitations should be acknowledged. First, the sample was derived predominantly from urban hospital centers, which may limit the generalizability of the findings to the entire Lebanese population, especially those in rural areas. While our case number is substantial, covering an estimated 20% of IBD patients nationwide, it is not a population-based registry of all cases. Second, the study’s retrospective design and reliance on existing medical records introduce the possibility of missing or incomplete data. We were unable to capture certain clinical and demographic details (for example, detailed medication history or socioeconomic factors) if they were not consistently recorded. Additionally, the analysis lacked longitudinal follow-up of patients; outcomes such as disease course and long-term complications could not be assessed, and incidence was inferred from diagnosis dates rather than observed in a prospective manner. Another limitation is the absence of data on environmental and lifestyle factors. Variables like smoking status, dietary habits, or degree of urbanization were not available in our dataset, preventing us from investigating their influence on IBD risk in this cohort. This is particularly relevant given that such factors are hypothesized to contribute to the rising incidence in transitioning countries. Finally, the scarcity of comparable studies from neighboring countries made it challenging to fully contextualize our findings in a Middle Eastern context. Differences in healthcare infrastructure and case-finding methods across countries might influence reported incidence and prevalence, so caution is needed when generalizing beyond Lebanon.

Despite these limitations, our study also has notable strengths. It is one of the largest and longest-running assessments of IBD epidemiology in Lebanon, spanning two decades and including nearly 3,000 patients. All cases were confirmed via histopathology and endoscopic evaluation, ensuring a high level of diagnostic certainty (indeed, no patients remained in an indeterminate colitis category). The breadth of data collected allowed us to characterize not only incidence and prevalence, but also demographic patterns and disease phenotypes in detail. Importantly, this work is among the first comprehensive epidemiological studies of IBD in the Lebanese and Arab context, helping to fill a gap in regional data. These strengths bolster the reliability of our findings and their relevance: the large sample size improves the precision of incidence/prevalence estimates, and the use of a centralized pathology database likely captured a consistent subset of cases over time. Taken together, the study’s strengths support its conclusions, while the acknowledged limitations highlight areas for cautious interpretation and future investigation.

### Recommendations for future research, policy and clinical practice

Looking ahead, our findings point to several key recommendations for research, public health policy, and clinical practice. Future research should build on this foundational data by addressing the gaps identified. Prospective, longitudinal studies are needed to track disease progression and outcomes over time, which would provide insight into the natural history of IBD in Lebanon (e.g., rates of surgery, complications, and response to therapies). Such studies should also incorporate the collection of environmental and lifestyle exposure data – for instance, evaluating the roles of smoking, diet, antibiotic use, and other factors – to better elucidate risk factors driving the rising incidence. Given the urban predominance of our sample, population-based registries or multi-center collaborations are recommended to ensure representativeness and allow regional comparisons.

Lebanon’s transition to a higher-incidence setting means that healthcare systems should strengthen capacities for IBD diagnosis and management. Policymakers and health authorities should consider strategic initiatives such as developing specialized IBD centers or clinics, training healthcare providers (gastroenterologists, surgeons, dietitians, and nurses) in up-to-date IBD care, and improving access to advanced therapies. Public health campaigns may also be warranted to raise awareness about IBD symptoms among the public and primary care providers, facilitating earlier referrals. The need for such measures is underscored by our finding that Lebanon is entering an acceleration phase of IBD epidemiology – a point at which timely intervention can help mitigate future burden. Investment in healthcare infrastructure (endoscopy units, pathology services, and medication supply chains) will be crucial to manage the growing number of cases effectively. Additionally, incorporating IBD into national health strategies (for chronic disease management or non-communicable disease programs) could ensure sustained attention to this condition as its prevalence climbs.

In clinical practice, awareness of the epidemiologic characteristics identified in this study can guide improvements in care. Clinicians should note the relatively young median age of IBD onset in Lebanon and maintain a high index of suspicion for IBD in younger patients presenting with chronic gastrointestinal symptoms. Early detection is critical for improving patient outcomes, and our data suggest that IBD is no longer rare in young adults. Knowledge of the predominant disease phenotypes (e.g., ileal Crohn’s disease, extensive ulcerative colitis) can aid clinicians in anticipating complications and tailoring therapy – for instance, recognizing that ileal CD may predispose to strictures or that extensive UC might require more intensive medical therapy or surgery. Our findings can also inform risk stratification and personalized management: for example, the slight male predominance and rising incidence mean clinicians should ensure both men and women are equally screened for IBD symptoms (countering. Ultimately, integrating these epidemiological insights – rising case numbers, patient demographics, and disease patterns – into clinical decision-making will help healthcare providers in Lebanon and similar settings to improve IBD care through earlier diagnosis and proactive management strategies.

## Conclusion

This study is among the first to provide data on incidence, prevalence and phenotypic characteristics in Lebanon and the Arab countries on the epidemiology of IBD. By highlighting patterns in disease onset, location, and demographic variations, the findings can guide clinicians in earlier detection, risk stratification, and personalized treatment strategies. The identification of Lebanon’s transition to the acceleration phase of IBD epidemiology underscores the need of strategic public health initiatives and enhanced healthcare resource allocation to manage the increasing disease burden effectively. This study establishes foundational data that future longitudinal studies in Lebanon and similar regions can build upon. By identifying baseline prevalence, it paves the way for further studies to track trends and causal relationships over time.
